# A 3-Month-Old Boy with a Giant Encephalocele—Resection of the Herniated Left Supra-Insular Hemisphere Without New Postoperative Motor Deficits

**DOI:** 10.3390/pediatric18040093

**Published:** 2026-07-10

**Authors:** Denis Ehrl, Vadym Burchak, Andrea Szelenyi, Joerg-Christian Tonn, Martin Staudt, Dorothee Rabenhorst, Mathias Kunz

**Affiliations:** 1 Department of Plastic, Reconstructive and Hand Surgery, Burn Centre for Severe Burn Injuries, Nuremberg Clinics, Paracelsus Medical University, 90419 Nuremberg, Germany; 2 Division of Hand, Plastic and Aesthetic Surgery, LMU University Hospital, LMU Medizin, Ludwig-Maximilians-Universität, 80336 Munich, Germany; 3 Department of Neurosurgery, Division of Pediatric Neurosurgery, LMU University Hospital, LMU Medizin, Ludwig-Maximilians-Universität, 81377 Munich, Germany; 4 German Cancer Consortium (DKTK), 81377 Munich, Germany; 5 Center for Pediatric Palliative Care, Department of Pediatrics, Dr. von Hauner Children’s Hospital, LMU University Hospital, LMU Medizin, Ludwig-Maximilians-Universität, 80377 Munich, Germany; martin.staudt@med.uni-muenchen.de (M.S.);

**Keywords:** congenital encephalocele, giant encephalocele, pediatric neurosurgery, corticospinal tract reorganization, hemispherectomy

## Abstract

**Background and Importance:** Congenital encephalocele refers to the protrusion of CNS tissue through a skull defect. As a rare neural tube malformation, clinical evidence is particularly limited for large encephaloceles, and management is subject to ongoing discussions. **Clinical Presentation:** A 3-month-old boy presented with a left hemisphere herniation above the level of the Sylvian fissure into a congenital parietal encephalocele. No focal deficits were appreciated. We hypothesized that early prenatal damage due to protruding brain tissue may have resulted in unihemispheric motor control of both body sides. As such, surgical repair guided by intraoperative electrophysiology and plastic reconstruction was scheduled. Intraoperatively, bilateral and symmetric extremity response upon transcranial electric stimulation of the contra-lesional right hemisphere was detected, whereas no responses from direct cortical and subcortical stimulation of the herniated brain parenchyma were elicited. Complete resection of the herniated supra-insular hemisphere was provided, and no ischemic changes or new deficits occurred. At 24-month follow-up, the patient showed voluntary movements with both upper extremities and voluntary grasping with his left (non-paretic) hand, no mirror movements, no signs of spasticity, good eye contact, and could speak several words. **Conclusions:** Safe resection with excellent outcome can be provided even for large encephaloceles. Intraoperative electrophysiological findings aid in identifying the absence of cortico-spinal projections and appear helpful to avoid post-operative deficits.

## 1. Introduction

The protrusion of brain tissue or meninges through a skull defect is denoted by the term encephalocele. While acquired encephalocele might be encountered in the setting of traumas or iatrogenic injuries, most cases are of a congenital nature due to incomplete separation of the different ectoderm layers after the closure of the neural folds. Incidence has been estimated at around 1 per 10,000 births [1,2]; however, large encephaloceles involving brain tissue are exceedingly rare, as such malformations are in utero detectable on screening ultrasound and may frequently result in pregnancy termination. Although early surgical reconstruction represents the standard of care for small encephaloceles [3], clinical management for large encephaloceles is less clear. Here, approaches range from resection of non-functional brain tissue over barrel stave osteotomies (allowing for proper skull growth around the herniated brain tissue) to supportive care only [4]. Surgical options depend crucially on the volume as well as on the function of the herniated brain.

## 2. Case Presentation

Screening ultrasound at 20 weeks of pregnancy in a third-time gravida yielded potential prolapse of fetal brain tissue through a skull defect with no further organ malformations being detected. After informed decision-making, the mother decided to deliver the baby via cesarean section at week 38, which was uncomplicated. A large encephalocele covered by skin was clinically seen. MRI confirmed the presence of brain tissue within the prolapsed sac, and the parents sought referral to specialized pediatric neurosurgical care.

**Figure 1:**
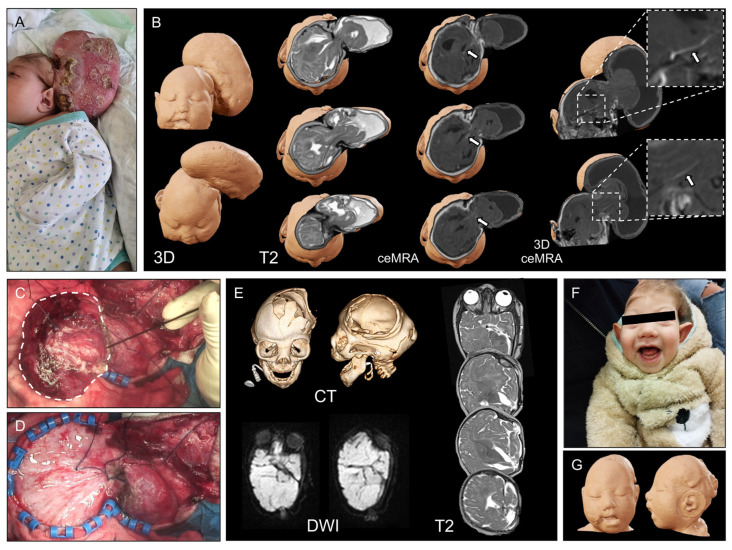
3-month-old with a giant left parietal encephalocele. (**A**,**B**) Congenital extrusion of the left supra-insular hemisphere supplied by the dominant M2 branch (arrows). (**C**,**D**) Intraoperative situs before (**C**) and after (**D**) resection of the herniated parenchyma (dotted line). (**E**–**G**) Postoperatively, CT demonstrates barrel stave osteotomy decompression, and MRI shows sufficient neurological and cosmetic outcome without infarcts on DWI.

As such, the 3-month-old boy was presented to our service with the left hemisphere herniating above the level of the Sylvian fissure into a congenital parietal encephalocele, for which vascular supply was provided by the dominant superior M2 division (Figure 1A,B). The brain was surrounded by fluid with restricted diffusion on MR imaging, corresponding to superinfected CSF resulting in empyema. No focal deficits, seizures, or signs of systemic inflammation were appreciated. Additional imaging-based screening showed a Chiari malformation type II with a cervicothoracic syringomyelia.

**Figure 2:**
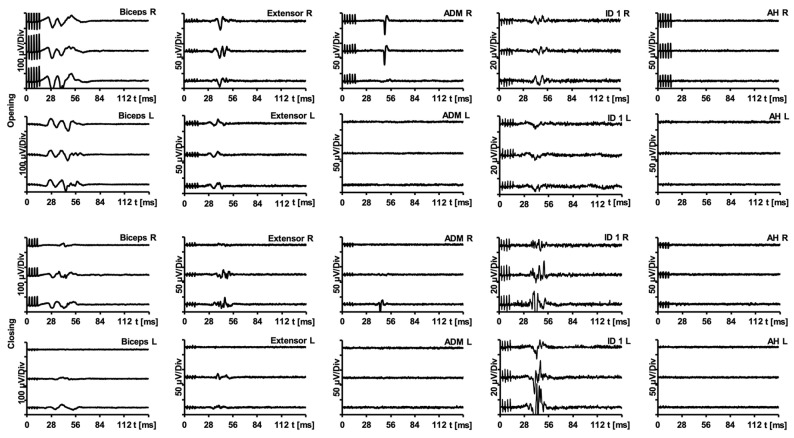
Intraoperative electrophysiological findings. Motor evoked potentials at the beginning (upper row) and end of the encephalocele resection (lower row) show response of the bilateral extremities upon stimulation. MEP amplitude decrease of both Biceps and Extensor and an increase of ID1 were considered to be related to brain shift following the resection of the encephalocele.

After extensive interdisciplinary discussion, a decision was made to first drain the superinfected fluid and provide antimicrobial coverage. An external ventricular drain catheter was placed via a small skin incision within the fluid, pus was evacuated, and targeted antibiotics were provided over three weeks. Repetitive imaging after removal of the catheter did not show recurrent or persistent signs of infection, and no new clinical symptoms occurred. Hoping for the existence of bilateral cortico-spinal projections from the contra-lesional hemisphere in the context of an extensive prenatal unilateral lesion, we scheduled the boy for surgical repair, including brain tissue resection guided by intraoperative electrophysiology and plastic reconstruction. Intraoperatively, bilateral and symmetric muscle responses from upper, but not lower extremity muscles were elicited by transcranial electric stimulation (maximum 220 mA, train-of-five methodology) of the contra-lesional right hemisphere whereas no responses from direct and sub-cortical stimulation (maximum 40 mA, anodal resp. cathodal monopolar probe stimulation; both following the standardized methodology as described by Sala et al., 2010 [5]) of the herniated brain parenchyma were generated(Figure 2). Such resection of the encephalocele was deemed possible, and complete resection of the herniated supra-insular hemisphere was achieved (Figure 1C,D). Barrel stave osteotomies were placed, and plastic reconstruction was achieved using a local fasciocutaneous flap after intraoperative perforator identification by transillumination (diaphanoscopy). No ischemic parenchymal changes, new deficits, or superinfection occurred (Figure 1E). Cosmetic results were excellent (Figure 1F,G). Radiological, clinical and neurodevelopmental examinations were thereafter performed in a 3-month interval to evaluate neurodevelopmental milestones and to exclude radiological signs of hydrocephalus, Chiari malformation and expansion of the syringomyelia. As part of ongoing follow-up examinations, at 24-month follow-up, the patient was happy, showed stable sitting with support and good head control (corresponding to a Gross Motor Function Classification System Level IV), good visual fixation, intermittent converging strabismus, voluntary movements with both upper extremities, and targeted grasping with his non-paretic left hand without mirror movements. No voluntary grasping with his (presumably paretic) right hand could be elicited. Muscle tone was normal, without signs of spasticity. He spoke several understandable words, responding to verbal cues. Homonymous right hemianopia must be expected radiologically; perimetry will be performed as soon as a sufficient level of cooperation is reached.

## 3. Discussion

Our case highlights that resection with excellent neurologic outcome can be provided even for large encephaloceles when the affected brain tissue is without cortico-spinal projections. Intraoperative electrophysiological findings were helpful to confirm such an assumption and therefore guide resection. Reportedly, in very young children, MEP can usually be elicited in at least one muscle [6,7]. Motor deficits, developmental diseases and the type of anesthesia might result in exceedingly increased motor threshold, resulting in absent MEP. As in our case, MEP in the lower extremity muscles is difficult to elicit. As such, the presented case gives another example that early prenatal damage may result in predominantly unihemispheric motor control of both body sides [8]. Notably, the corticospinal tract is embryologically of bilateral origin, and, in healthy brains, activity-dependent elimination of ipsilateral projections takes place peri- and postnatally [9]. Extensive unilateral brain malformations frequently lead to reorganization in the motor system with ipsilateral projections from the contra-lesional hemisphere [10,11], and hemispherotomies in such patients can be performed without losing the grasping ability of the contralateral hand [12]. Hence, it is tempting to speculate that similar mechanisms were also involved in the presented case. Given the limited follow-up, we cannot comment on any long-term deficits, including cognitive outcomes. In this context, informed decision-making together with the parents and interdisciplinary involvement of dedicated medical specialties appears to be of utmost importance to highlight the range of uncertainty even when the best neurosurgical care is provided.

Large or so-called giant encephaloceles—conventionally defined as a herniation sac larger than the patient’s head—are exceedingly rare, and the available evidence consists almost entirely of single case reports and small series [4,13,14]. The largest systematic review to date assembled only 74 surgically treated giant occipital encephaloceles reported between 1959 and 2021, with a mean age at operation of 3.5 months and an overall postoperative survival of approximately 90% [13], whereas a recent three-centre cooperative study of 79 posterior-vault encephaloceles confirmed that parietal lesions are frequently accompanied by vascular anomalies and that large series with documented long-term outcomes remain scarce [14]. Across these reports, the most consistently identified determinants of outcome are the presence of hydrocephalus, the amount of herniated brain tissue, and an associated syndromic or malformative context, whereas the anatomical location of the defect has been reported less consistently as an independent predictor [2,13,15,16]. The reported management strategies span a broad spectrum, ranging from resection of non-functional herniated tissue, through staged reduction and reconstruction with preservation of the parenchyma, to barrel-stave osteotomies that accommodate the herniated brain and, at the most severe end, supportive or palliative care [4,14,15].

Our patient differs from most previously reported cases in several respects (Table 1). First, the majority of published giant encephaloceles are occipital, whereas the present lesion was parietal and contained the entire supra-insular portion of one cerebral hemisphere rather than occipital or cerebellar tissue. Second, in contrast to reports in which the herniated tissue is preserved and reduced intracranially [14,15] or in which an antenatal prognosis is judged to be dismal, we elected to resect the herniated hemisphere only after intraoperative electrophysiology had demonstrated the absence of cortico-spinal projections from the malformed tissue. The recent case reported by Spoor and colleagues is instructive in this regard: an infant with a giant parieto-occipital encephalocele in whom an initial palliative plan was revised in favor of surgical reconstruction at four weeks of age subsequently showed substantial developmental progress in speech, language and motor skills at four years of follow-up [14]. Together with our case, this illustrates that an unfavorable antenatal prognosis does not invariably preclude a good functional result and underscores the value of individualized, function-based decision-making.

From a technical standpoint, the present case adds to the small body of evidence supporting intraoperative neurophysiology as a decision-making tool in encephalocele surgery. Whereas the decision to resect or preserve herniated parenchyma has traditionally rested on imaging and intraoperative inspection, the combination of transcranial electrical stimulation of the healthy hemisphere with direct cortical and subcortical mapping of the malformed tissue allowed an objective, real-time assessment of whether the herniated hemisphere contributed to motor control [17]. Positive cortical/subcortical responses on the herniated left hemisphere or missing responses elicited ipsilaterally on the right hemisphere would, in all likelihood, have led to a change in the surgical strategy toward a more conservative approach, with less resection and more bony reconstruction. The absence of any motor response from the herniated parenchyma, together with preserved bilateral responses from the contra-lesional hemisphere, provided the physiological rationale for safe complete resection. Several limitations must nevertheless be acknowledged. This is a single case, and the follow-up of 24 months, although informative for early motor milestones, is too short to characterize cognitive, language and visual outcomes, which we therefore plan to reassess at school age. The absence of an intraoperative motor response does not formally exclude a contribution of the resected tissue to non-motor functions, and electrophysiological findings should always be interpreted within the broader clinical and imaging context. Finally, generalizability is inherently constrained by the rarity and heterogeneity of large encephaloceles, reinforcing the importance of multidisciplinary, individualized decision-making and of transparent counseling of families regarding residual uncertainty.

## 4. Conclusions

Neurosurgical repair with a favorable outcome can be provided even for large encephaloceles. Intraoperative electrophysiological findings aid in identifying brain tissue without cortico-spinal projections and appear helpful to avoid post-operative deficits.

## Figures and Tables

**Table 1:** Comparison of the present case with representative reported large or giant encephaloceles containing brain tissue. Aggregate figures from a systematic review and a multicentre series are included for context. EVD, external ventricular drain; IONM, intraoperative neurophysiological monitoring; M2, second (insular) segment of the middle cerebral artery.

Study (Year) [Ref]	Age at Surgery; Sex	Location and Herniated Contents	Associated Features	Management	Outcome (Follow-Up)
Present case (2026)	3 months; male	Left parietal; entire supra-insular cerebral hemisphere, supplied by the dominant M2 branch	Chiari type II malformation; cervicothoracic syringomyelia; infected sac (empyema)	External ventricular drainage and antibiotics for infection, then IONM-guided complete resection of the herniated hemisphere; barrel-stave osteotomies; local fasciocutaneous flap	No new deficit; at 24 months, bilateral voluntary upper-limb movement, left-hand grasp, no spasticity, several spoken words; residual right hemiparesis
Dronkers et al. (2026) [14]	4 weeks; infant	Parieto-occipital; occipital and superior parietal lobes and superior cerebellum	Absent vein of Galen; hypoplastic dural sinuses	Surgical reconstruction (reduction and repair) after an initial palliative plan was revised	Substantial developmental progress (speech, language, motor) at 4 years
Yindeedej et al. (2023) [13]; systematic review (74 cases)	Mean 3.5 months	Occipital (giant; larger than the head)	Microcephaly, callosal agenesis and Chiari malformation common	Excision with or without duroplasty/reconstruction (various strategies)	Survival reported in 64 patients (90.1%); postoperative complications in 14 cases (16 events)
Pasquali et al. (2025) [15]; cooperative series (79 cases)	Series of 79; age at surgery not individually reported	Posterior vault (46 parietal, 33 occipital)	Vascular anomalies frequent in parietal (88%); cerebral anomalies more common in occipital (45% vs. 15%)	Surgical correction in all patients	Psychomotor impairment in 54% of occipital vs. 17% of parietal cases (mostly mild in parietal); 3 occipital deaths
Schneider et al. (2023) [4]	Infancy	Large vertex	Variable associated anomalies	Reduction/resection with cranial reconstruction	Management and outcomes reviewed; generally favourable with appropriate selection

## Data Availability

The original contributions presented in this study are included in the article. Further inquiries can be directed to the corresponding authors.
